# Impact of Ventilatory Modes on the Breathing Variability in Mechanically Ventilated Infants

**DOI:** 10.3389/fped.2014.00132

**Published:** 2014-11-25

**Authors:** Florent Baudin, Hau-Tieng Wu, Alice Bordessoule, Jennifer Beck, Philippe Jouvet, Martin G. Frasch, Guillaume Emeriaud

**Affiliations:** ^1^Department of Pediatrics, CHU Sainte-Justine, Université de Montréal, Montreal, QC, Canada; ^2^Department of Mathematics, University of Toronto, Toronto, ON, Canada; ^3^Pediatric Critical Care Unit, Geneva University Hospital, Geneva, Switzerland; ^4^Keenan Research Centre for Biomedical Science, Li Ka Shing Knowledge Institute, St. Michael’s Hospital, Toronto, ON, Canada; ^5^Department of Pediatrics, University of Toronto, Toronto, ON, Canada; ^6^Department of Obstetrics and Gynecology, CHU Ste-Justine Research Center, Université de Montréal, Montreal, QC, Canada; ^7^Department of Neurosciences, CHU Ste-Justine Research Center, Université de Montréal, Montreal, QC, Canada; ^8^Centre de recherche en reproduction animale, Université de Montréal, St-Hyacinthe, QC, Canada

**Keywords:** pediatric intensive care, mechanical ventilation, neurally adjusted ventilatory support, diaphragm, children

## Abstract

**Objectives**: Reduction of breathing variability is associated with adverse outcome. During mechanical ventilation, the variability of ventilatory pressure is dependent on the ventilatory mode. During neurally adjusted ventilatory assist (NAVA), the support is proportional to electrical activity of the diaphragm (EAdi), which reflects the respiratory center output. The variability of EAdi is, therefore, translated into a similar variability in pressures. Contrastingly, conventional ventilatory modes deliver less variable pressures. The impact of the mode on the patient’s own respiratory drive is less clear. This study aims to compare the impact of NAVA, pressure-controlled ventilation (PCV), and pressure support ventilation (PSV) on the respiratory drive patterns in infants. We hypothesized that on NAVA, EAdi variability resembles most of the endogenous respiratory drive pattern seen in a control group.

**Methods**: Electrical activity of the diaphragm was continuously recorded in 10 infants ventilated successively on NAVA (5 h), PCV (30 min), and PSV (30 min). During the last 10 min of each period, the EAdi variability pattern was assessed using non-rhythmic to rhythmic (NRR) index. These variability profiles were compared to the pattern of a control group of 11 spontaneously breathing and non-intubated infants.

**Results:** In control infants, NRR was higher as compared to mechanically ventilated infants (*p* < 0.001), and NRR pattern was relatively stable over time. While the temporal stability of NRR was similar in NAVA and controls, the NRR profile was less stable during PCV. PSV exhibited an intermediary pattern.

**Perspectives**: Mechanical ventilation impacts the breathing variability in infants. NAVA produces EAdi pattern resembling most that of control infants. NRR can be used to characterize respiratory variability in infants. Larger prospective studies are necessary to understand the differential impact of the ventilatory modes on the cardio-respiratory variability and to study their impact on clinical outcomes.

## Introduction

Breathing is a cyclic activity with inspiratory and expiratory phases, which is not monotonous (1–3). Priban (4) has shown in 1963 that respiration is extremely variable. While it is almost impossible to observe two spontaneous breaths with exactly the same characteristics, breathing is not random either. Respiratory variability is an intrinsic property of breathing and reflects the degree of freedom of the respiratory control system (5, 6). A low respiratory variability is associated with pathological conditions in adults (7, 8) and in infants (9). During mechanical ventilation, respiratory variability alteration may have an important impact on alveolar recruitment, oxygenation, and diaphragmatic dysfunction (10–14). A low respiratory variability is predictive of mechanical ventilation weaning failure (15–17) and mortality (18).

Neurally adjusted ventilatory assist (NAVA) is a recent ventilatory mode (19) during which the assist pressure is proportional to the electrical activity of the diaphragm (EAdi), which directly reflects the activity of the neural respiratory command (20, 21). In contrast to monotonous ventilation delivered by more conventional ventilatory modes, such as pressure-controlled ventilation (PCV), pressure support ventilation (PSV), or volume-controlled ventilation (VCV), the variability of pressure and tidal volume is higher during NAVA (11, 22–24). NAVA permits to transmit the variability of the respiratory center demand into pressure (and volume) variability (11, 22, 23). While different ventilatory modes have markedly different impact on the variability of the ventilatory pressure or flow, the impact of these modes on the patient’s own breathing activity and variability is not clear (11, 23). In a study conducted in 10 infants, we observed similar coefficients of variation of inspiratory EAdi during NAVA ventilation as compared to PCV and PSV, while the coefficients of variation for ventilatory pressure were strikingly different (23). Assuming that coefficients of variation may be insufficient to capture the non-linear properties of the signal at different time scales and hence fail to discriminate the groups, we sought to investigate the effects of NAVA on endogenous respiratory drive using more advanced techniques capturing variability.

The synchrosqueezing transform method, and in particular the related non-rhythmic to rhythmic (NRR) index, has recently emerged as a new method to analyze respiratory variability with a great robustness to noise and to short duration of evaluation period (25–27).

The aim of this study was to characterize the variability of the respiratory center activity (reflected by EAdi) in infants using NRR, and to assess the impact of different ventilatory modes on the variability pattern. We hypothesized that on NAVA, EAdi variability will resemble most the endogenous respiratory drive patterns seen in the control group.

## Materials and Methods

This retrospective analysis included patients from two previous studies performed in the pediatric intensive care unit (PICU) of Sainte-Justine Hospital, Montreal, Canada. One study was conducted during mechanical ventilation with three different ventilatory modes (23) and one study was conducted in infants spontaneously breathing after tracheal extubation (28). This *post hoc* analysis (Number # 3959) and the two previous studies (# 2537 and 3113) were approved by the Ethics Committee of Sainte-Justine Research Center. Written informed consent was obtained from the parents or guardian prior to inclusion in the initial studies.

### Patients

In the two studies, children <12 months old admitted to the PICU and requiring invasive mechanical ventilation for more than 24 h were eligible. For both studies, the exclusion criteria were chronic respiratory insufficiency, tracheostomy, pneumothorax, degenerative neuromuscular disease, bleeding disorders, vasoactive drug infusion, cyanotic congenital cardiovascular disease, diagnosed phrenic nerve damage, esophageal perforation, high frequency oscillatory or jet ventilation, contraindication to change nasogastric tube, and parental refusal.

### Study protocol

Electrical activity of the diaphragm signal was recorded using a specific nasogastric tube (NAVA catheter, Maquet, Solna, Sweden) and a dedicated Servo I ventilator (Maquet, Solna, Sweden) as previously described (23, 28–30). The ventilatory pressure was simultaneously recorded from the ventilator in mechanically ventilated patients. The sampling rate was 62 Hz for both signals.

In the mechanical ventilation group, infants (*n* = 10) were recorded consecutively in three ventilatory modes: NAVA for 5 h, PCV for 30 min, and PSV for 30 min (23). The last 10 min in each mode were analyzed; the three consecutive recordings were, therefore, obtained within a period lasting about 70 min. A longer phase of ventilation was obtained with NAVA because this was one of the first studies evaluating NAVA in children, and we wanted to observe the behavior during this mode for several hours.

In the spontaneously breathing group (control group), infants (*n* = 11) who had recovered from a mechanical ventilation period were recorded during spontaneous ventilation in stable conditions, i.e., in the 2 h prior to PICU discharge, or when the removal of the EAdi catheter was planned because respiratory status was normal. The median (interquartile) time between extubation and recording was 24 [12–36] h. No data on ventilatory pressure were available in this group, since no ventilatory support was provided.

### Variability analysis

The NRR index (25) was used to describe the variability of EAdi and ventilatory pressure signals based on the synchrosqueezing transform. The power spectrum and its underlying mathematical model do not take into account the momentary behavior of the oscillatory pattern of a given signal (31, 32). For example, the variability of the momentary breathing rate is ignored in the power spectrum analysis. This fact renders the power spectrum unsuitable for analyzing variability of EAdi signals under the control of different ventilator modes. Synchrosqueezing transform is a modern signal processing technique aiming to capture this momentary behavior – the quantities *amplitude modulation* and *instantaneous frequency* in the model analyzed by the synchrosqueezing transform capture how large/small and how fast/slow the periodic pattern inside EAdi signals repeats itself at each observation moment. We call a periodic pattern with slowly varying amplitude modulation and instantaneous frequency *rhythmic*, and the other patterns *non-rhythmic;* in other words, if the periodic pattern changes slowly as time goes by, we call the signal rhythmic. The NRR index quantifies how rhythmic the signal is; the higher the NRR index is, the more variable the signal is. We refer the reader to Ref. (25) for details of NRR and Ref. (26, 33) for the theoretical derivation of these indices.

As the optimal time window to analyze respiratory signals with NRR is not established and taking advantage of NRR’s ability to estimate variability at relatively short time scales, we calculated the NRR using two time scales: 10 min (the whole recorded period) and 2 min (rendering 5 intervals per patient). For each patient, the mean value and the standard deviation (SD) of the five 2 min period NRRs were calculated. The stability of the 2-min NRR over time was estimated with intra-patient coefficients of variation (SD divided by the mean).

### Statistical analysis

Group data are reported as median [25th–75th percentiles] unless otherwise specified. All statistical analyses were conducted using SPSS software (Version 22, IBM SPSS Statistics, IBM Corporation, Armonk, NY, USA). Generalized estimating equations (GEE) modeling approach was used to assess the effects of different modes of ventilation while accounting for time effect and Paw (34). We used a linear scale response model with time and ventilation group as predicting factors and pressure variability as a covariate to assess their main effects and interactions using maximum likelihood estimate and Type III analysis with Wald Chi-square statistics. Friedman’s test or Wilcoxon’s test were used to assess differences between ventilatory modes (non-parametric paired sample). A *p*-value below 0.05 was considered significant.

## Results

### Population characteristics

Ten infants were analyzed in the mechanical ventilation group and 11 infants in the control group. For the entire population, the median age was 3 [1–5] months and the weight 4.8 [3.7–5.9] kg. The main characteristics of each group are presented in Table 1. The baseline characteristics were similar among the groups except for smaller body weight in the control group (*p* = 0.04).

**Table 1:** **Baseline characteristics of the patients in the mechanical ventilation and the control groups**.

	Mechanical ventilation (*n* = 10)	Control (*n* = 11)
Age (months)	4.5 [2.5–4.7]	1.5 [1–3]
Weight (kg)	5.7 [4.8–6.7]	3.9 [3.5–5.0]^a^
Male gender	4 (40)	4 (36)
Admission FiO_2_	0.35 [0.30–0.39]	0.35 [0.30–0.35]
Admission diagnosis	
Bronchiolitis	3 (30)	5 (45)
Pneumonia	1 (10)	2 (18)
Post surgery	4 (40)	1 (9)
Sepsis	0 (0)	2 (18)
Other	2 (20)	1 (9)

*Data are expressed as median [25th–75th percentile] or number (percentage)*.

*^a^*p* < 0.05*.

The clinical status and the ventilatory settings in the different recording conditions are detailed in Table 2. The control group patients had a higher respiratory rate than mechanically ventilated infants (*p* < 0.05). The peak EAdi was significantly lower in PCV as compared to NAVA and control groups (*p* < 0.05).

**Table 2:** **Clinical and ventilatory characteristics during the recordings**.

	Mechanical ventilation group			Control group
	NAVA	PCV	PSV	
Clinical parameters			
Heart rate, bpm	130 [128–140]	131 [129–143]	126 [118–141]	147 [135–165]
SpO_2_,%	100 [99–100]	99 [99–100]	100 [100–100]	99 [98–100]
Ventilatory parameters			
Inspiratory pressure, cmH_2_O	18 [14–20]	16 [15–17]	16 [15–20]	NA
PEEP, cmH_2_O	5 [5–5]	5 [5–5]	5 [5–5]	NA
Tidal volume, ml/kg^−1^	8.5 [7.3–11.4]	7.3 [6.9–8.5]	8.3 [6.8–11.5]	NA
Peak EAdi, μV	15.6 [5.6–18.2]	7.1 [4.3–10.5]^a^	6.3 [4.8–15.6]	18.0 [11.6–23.4]
EAdi respiratory rate, bpm	43 [40–49]	38 [37–41]	48 [41–54]	72 [62–80]^b^

*Data are reported as median [25th–75th percentile]*.

*SpO_2_, Oxygen saturation; PEEP, positive end-expiratory pressure; EAdi, electrical activity of the diaphragm*.

*^a^Significant difference between PCV and control and between PCV and NAVA (*p* < 0.05)*.

*^b^Significant difference between control and each ventilatory mode (*p* < 0.05)*.

### Respiratory variability

**Figure 1:**
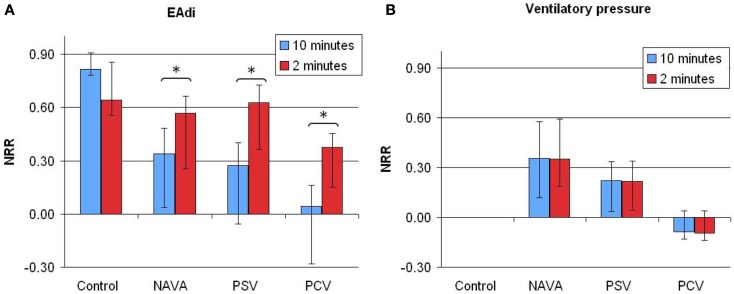
**Non-rhythmic to rhythmic (NRR) index for electrical activity of the diaphragm [EAdi (A)] and ventilatory pressure (B) signals, calculated over 10 min (blue bars) or 2 min (red bars) periods in infants without ventilatory support (control) and during mechanical ventilation in neurally adjusted ventilatory assist (NAVA), pressure support ventilation (PSV), and pressure-controlled ventilation (PCV)**. Note that time scale of assessing variability using NRR index has an effect on estimating EAdi variability (*p* = 0.03), but not on estimating the ventilator pressure variability (*p* = 0.44). NRR, arbitrary units. Data are presented as median [25–75%]. *p < 0.01 in pairwise comparison.

The NRR indices for EAdi and ventilatory pressure in the different ventilatory conditions are reported in Figure 1.

#### Pressure variability

The pressure variability NRR was higher during NAVA than during PCV (0.36 [0.11–0.58] vs. −0.08 [−0.12–0.04]; *p* = 0.013), reflecting a higher proportion of NRR components, i.e., an increased variability. The difference between PSV and NAVA for pressure variability was not significant (*p* = 0.11). There was no time scale effect on the pressure variability analysis with NRR: the NRR indices calculated from 2-min or 10-min periods were similar.

#### EAdi variability

On 10-min time scale, the NRR for EAdi signals were higher in the control group infants as compared to patients with mechanical ventilation (Figure 1, *p* < 0.0001, with significant differences between control and each ventilatory mode). No significant difference was observed between the three ventilatory modes (*p* = 0.40).

**Figure 2:**
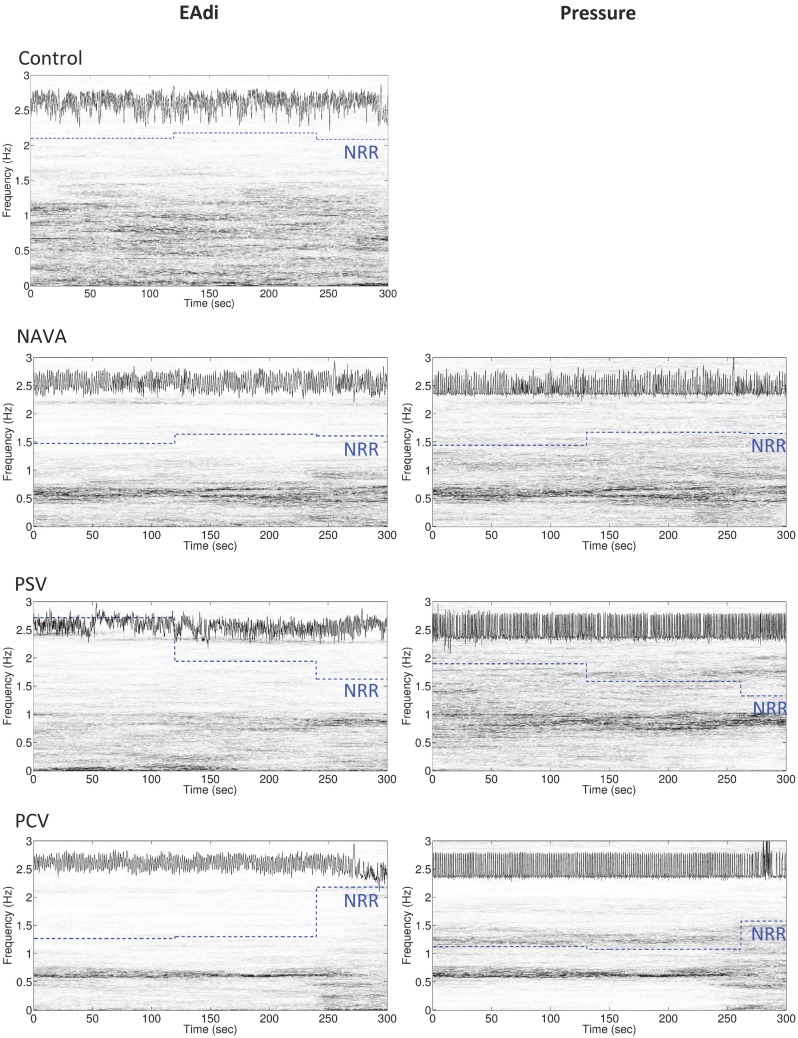
**Representative example of the variability of non-rhythmic to rhythmic (NRR) index for electrical activity of the diaphragm (EAdi, left panels) and pressure (right panels) over 5 min in an infant during mechanical ventilation in neurally adjusted ventilatory assist (NAVA), pressure support ventilation (PSV), and pressure-controlled ventilation (PCV), and in a spontaneously breathing infant (control, with only EAdi signal)**. In each panel, the original signal is displayed in the upper part of the box (the signal on the EAdi column is the log 10 of the original EAdi signal), the time-varying power spectrum (the time–frequency representation determined by synchrosqueezing transform) is continuously represented on a vertical axis (gray distribution), and the piecewise constant blue dotted lines represent the NRR shifted up by 1.3 for the corresponding 2 min intervals. Note that the more rhythmic the oscillation is, the smaller the NRR value becomes. Also note the change in power spectra of both pressure and EAdi at the end of the PCV recording, which is translated into an increase in NRR.

**Figure 3:**
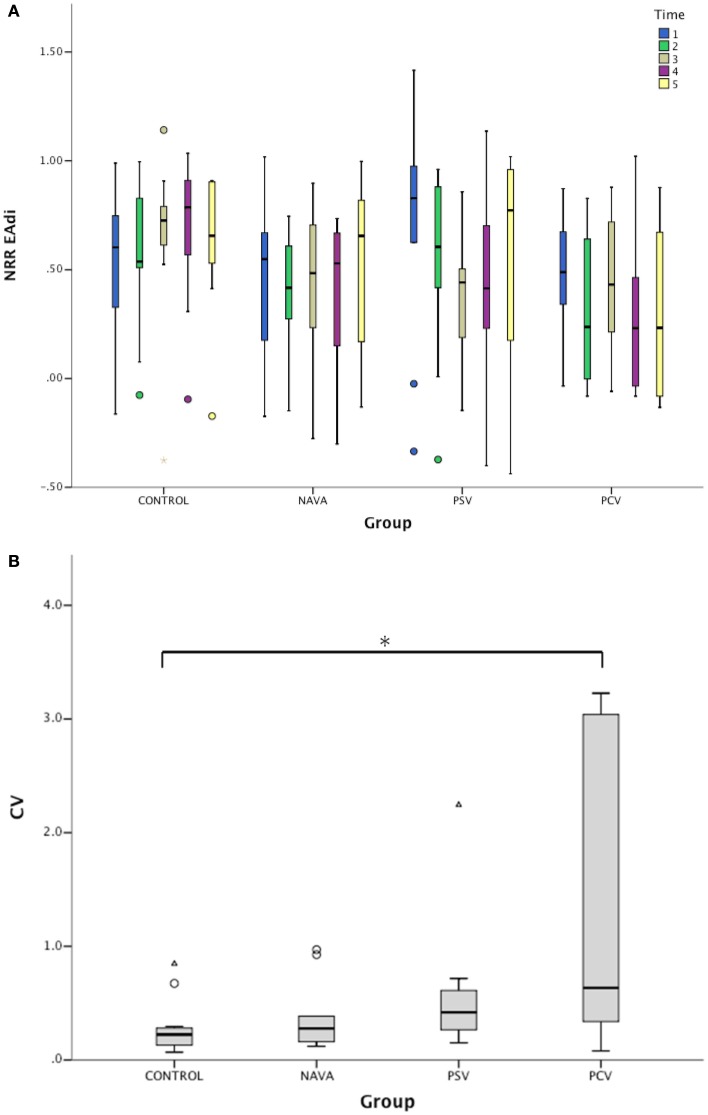
**(A)** Variation of non-rhythmic to rhythmic (NRR) index during the five consecutive 2-min periods for electrical activity of the diaphragm (EAdi) signal in infants without ventilatory support (control) and during mechanical ventilation in neurally adjusted ventilatory assist (NAVA), pressure support ventilation (PSV), and pressure-controlled ventilation (PCV). NRR EAdi, arbitrary units. **(B)** Corresponding intra-patient coefficients of variation (CV) of NRR for EAdi signal. Median [25–75%]. *p < 0.05 vs. control.

On 2-min time scale, the impact of the ventilatory condition was less apparent (Figure 1). However, the 2-min windows revealed intra-individual temporal variability of NRR. A representative example of variability profile and NRR changes over time for ventilatory pressure and EAdi is illustrated in Figure 2. The pattern of NRR variability for EAdi for the group is provided in Figure 3, with statistical summary.

No correlation was observed between the age and NRR calculated on 10- or 2-min periods in the entire study group, as well as in the control group only (all *R*^2^ < 0.02, all *p* > 0.7).

Generalized estimating equations showed that the time, the interaction between ventilatory mode and time, and the interaction among ventilatory mode, time, and pressure NRR were significant factors predicting EAdi NRR (*p* < 0.0001, Table 3; Figure 3A). The impact of the ventilatory modes in the aforementioned interaction was significant in each case (i.e., NAVA vs. PSV, NAVA vs. PCV, and PSV vs. PCV, all *p* < 0.001).

**Table 3:** **Generalized estimating equations model for NRR EAdi estimated on 2-min time scale**.

Variables	Wald chi-square	df	Significance
(Intercept)	31.8	1	<0.001
Time	15.2	4	<0.005
Ventilatory mode	3.2	2	0.198
Time * Vent. mode	2020	8	<0.001
Time * Vent. mode * NRR pressure	582.8	9	<0.001^a^

*Vent. mode, ventilatory mode; df, degree of freedom*.

*^a^In subgroup comparisons, this interaction was significant for each between group comparison, i.e., NAVA vs. PSV, NAVA vs. PCV, and PSV vs. PCV (all *p* < 0.001)*.

The intra-individual temporal NRR variability was also quantified using coefficients of variation of NRR, which were affected by the ventilatory condition (*p* < 0.01, Figure 3B). While the variability of NRR was low in the control group (CV 22% [13–28]) and during NAVA (28% [27–60], *p* = 0.33 vs. control), the CVs were 42% [27–60] during PSV (*p* = 0.09 vs. control), and 63% [35–279] during PCV (*p* = 0.02 vs. control).

## Discussion

Our results confirm that the mechanical ventilation influences the variability of the respiratory command. Normally breathing infants exhibited relatively high respiratory variability (as assessed by NRR), but this variability pattern seems rather stable over time. All ventilatory modes were associated with lower variability as compared to non-intubated patients. While the stability of NRR was similar in NAVA as compared to controls, the variability pattern was less stable during PCV, and PSV exhibited an intermediary pattern. Moreover, this physiological study is the first to evaluate respiratory variability using NRR in a pediatric population.

Physiological variability is an essential property of living systems that allows the adaptation to internal and external constraints. The variability pattern reflects this adaptability. The loss of variability usually reflects a loss in the degrees of freedom of the complex system. Decreased respiratory variability in critically ill adult patients has been shown to be predictive of poor outcome (15, 17) and even mortality (18). Variability is not random and seems to be organized around a physiological balance (11). For this reason, we decided to compare the respiratory variability of ventilated children to a control group. This allowed us to appreciate baseline physiological fluctuations in normal breathing pattern and to evaluate the impact of mechanical ventilation.

The mechanical ventilation has an impact on the patient’s breathing. In particular, the ventilatory assist can elicit the Hering Breuer reflex resulting in prolonged expiration, or interrupted inspiratory time (35). The ventilatory support also influences the magnitude of the patient’s own respiratory effort through a negative feedback (28, 36, 37). The impact of mechanical ventilation on the variability pattern of respiration has seldom been studied in comparison to non-supported patients. We previously observed in neonates that mechanical ventilation was associated with a markedly blunted variability of the functional residual capacity (9). In the present study, we confirm that mechanical ventilation decreases the respiratory variability, as illustrated by the lower NRR component in the EAdi signals in the three ventilatory modes as compared to control infants.

Due to the relatively monotonous ventilatory modes commonly deployed in intensive care, and in light of the adverse outcomes associated with decreased respiratory variability, the impact of the ventilatory modes on the variability has been a matter of concern. Biologically variable ventilation has been developed in order to artificially reintroduce variability in the ventilatory volumes and timing. Experimental data suggest that it could permit to improve lung recruitment and oxygenation, but the experience in clinical practice is very limited (13, 38). The NAVA mode is a recently introduced ventilatory mode that also has the potential to improve variability. Indeed, in NAVA, the ventilator delivers a pressure support that is synchronized and proportional in amplitude with EAdi. EAdi is a reliable reflection of the ventilatory demand of the respiratory center, and it contains a natural variability (20). Under NAVA, the EAdi variability is translated into variability of ventilatory pressure and timing. This theoretical concept has been confirmed in adults (11, 22) and in infants (23, 24). While it is clear that the variability in ventilatory pressure and timing is improved with NAVA as compared to the more monotonous ventilatory modes (11, 22–24), the impact on the patient’s own respiratory pattern is less clear. In critically ill adult patients, Schmidt et al. (11) characterized the variability pattern of ventilatory flow and EAdi in NAVA and PSV. While they confirmed an increased variability and complexity of flow in NAVA, they did not find any difference for EAdi variability pattern. Delisle et al. (22) also described the variability pattern of volume, flow, and EAdi during NAVA and PSV in adults. Based on the coefficients of variation, they reported increased variability pattern for volume and flow with NAVA. However, the coefficients of variation for EAdi were superior in PSV during the sleep stage 1–4 (non-REM), and showed no difference in REM sleep. Interestingly, during non-REM sleep phases, PSV was associated with a higher incidence of apneas, and an oscillatory pattern with episodes of over-assistance followed by apneas was observed in PSV. This may explain the increased coefficients of variation of EAdi in PSV during these periods, while in NAVA the intrinsic feedback prevented over-assistance and apneas, and the coefficient of variation was lower. This illustrates the equilibrium that should be targeted with sufficient but not excessive variability. In pediatric patients, ventilatory pressure and volume were found more variable during NAVA than PSV or PCV (23, 24). However, no difference in EAdi variability pattern was observed in these pediatric studies, based on the coefficient of variation.

On the theoretical basis that the patient’s respiratory centers are exposed to different feedback depending on the ventilatory mode and its delivered pressure variability and well-known non-linear nature of the respiratory activity (11), we hypothesized that the non-linear properties of the breathing pattern were differentially influenced, i.e., in a way that was not tracked by the coefficients of variation. We, therefore, used the NRR index to describe the respiratory variability. NRR is based on synchrosqueezing transform, which is a novel time–frequency analysis technique originally introduced in order to analyze speech signals. With synchrosqueezing transform, instantaneous frequency and the amplitude modulation can be accurately estimated from relatively short time intervals and synchrosqueezing transform is robust to different types of noise (27). NRR index captures the temporal dynamics of the respiratory oscillations. In addition to the rhythmicity as the key ingredient, NRR also captures another local information hidden inside the signal, for example, how breathing evolves from one cycle to the next. In a nutshell, it takes into account not only the instantaneous frequency and the amplitude modulation but also the cycle to cycle temporal evolution. Instantaneous frequency and the amplitude modulation were previously used to predict weaning success in adult with a ROC area under curve of 0.76 and with only 3 min of respiratory data when conventional analysis tools required more than 30 min signal (27). NRR index was also applied to evaluate the heart rate variability, which was shown to be well correlated with the anesthesia depth and predicted well the first response after the termination of anesthesia (25). In the present study, the NRR analysis confirmed the overall lower variability of EAdi during mechanical ventilation as compared to the control group. While NAVA seems associated with a higher NRR, the difference among the ventilatory modes was not significant and larger studies will be needed to draw definitive conclusions. Importantly, we observed that time scale of observation is an important factor in estimating NRR of EAdi variability. No optimal time scale is known *a priori*. While the 10-min interval is helpful in assessing a global pattern and probably better captures the temporal evolution aspects of the respiratory variability reflected in the NRR, shorter time intervals permit to study the fluctuations in the EAdi variability pattern, as illustrated in Figure 2. We observed that during normal breathing, NRR indices were relatively high but exhibited little variations (i.e., low coefficient of variation of NRR). This means a relatively “regular variability.” Interestingly, these temporal NRR fluctuations were similarly small in NAVA. Contrastingly, PCV was associated with relatively low NRR and higher coefficient of variation of NRR, thereby reflecting an “irregular variability.” A trend for higher coefficient of variation of NRR on PSV (*p* = 0.09 as compared to controls) suggests that the variability on PSV could also be more irregular than in controls and on NAVA. This finding parallels the observations by Delisle et al. (22) and requires larger cohorts to be validated.

The reduction of the perturbation of the ventilatory drive may have potential clinical benefits, which should be assessed in future studies. This may decrease the incidence of apneas or hypoventilation episodes (22, 39), improve the patient’s comfort during ventilation, and ameliorate the quality of sleep (22) in critically ill children.

Our study has several limitations. It is a retrospective study based on *post hoc* analysis. The duration of the recordings was relatively short and the patient sample size was small, in line with this being a pilot study. The sleep status was not recorded. The patients were selected from two previous studies with similar inclusion criteria, but the two groups were slightly different. In particular, the control group patients tended to be younger, although this difference was not statistically significant (*p* = 0.07). The younger age may be an important factor as it can be associated with a relatively more periodic breathing, which could influence our variability analysis. Although we did not observe any association between age and NRR, we cannot exclude that this has been a confounding factor. The control group included spontaneously breathing infants with no need for ventilatory support, but they had recovered from a period of mechanical ventilation in PICU. They should not be considered “healthy controls,” but rather represent stable recovering patients. The mechanically ventilated group included patients able to maintain spontaneous ventilation together with their ventilatory assist. The results do not reflect the conditions of patients deeply sedated or with a full ventilatory support. Reflecting the usual PICU patients, the population in this study was somewhat heterogeneous with a variety of clinical diagnoses. This heterogeneity may have diluted the effect of the ventilatory modes on the breathing variability, provided the response to the ventilatory mode depends on the patient’s condition. The limited sample size did not permit to conduct subgroup analysis, which requires future investigations in prospective cohorts. After the ventilatory mode changes, a 20 min “washout” period was allowed before analyzing the respiratory variability. The optimal duration for reaching an equilibrium is not known, although the change in variability pattern appears extremely rapid (a few seconds) in clinical practice. Other studies have used 10 min “washout” periods in adults (11) as in infants (24). Of note, relatively similar findings on respiratory variability have been observed using periods of 4 h (22) or 10 min (11). Using 20 min period permitted a balance between the time to equilibrate and the total study duration.

Importantly, although the association between the loss of variability and adverse outcome has been repeatedly reported (15, 17, 18), a confounding association with the underlying pathology is highly possible, as discussed above. Only studies with interventional design will permit to assess the clinical impact of variability restoration.

## Conclusion

Non-rhythmic to rhythmic permits to characterize the variability pattern of the respiratory drive in infants with or without ventilatory support. In normally breathing infants, NRR was higher and with little variation, as compared to mechanically ventilated infants. However, this finding should be considered as exploratory, as it is based on a *post hoc* analysis and some baseline characteristics differed between the two groups. Although NAVA seemed to have the smallest impact on the variability pattern of the ventilatory demand, the differences with the other modes reached significance on some, but not all time scales of observation. Further studies are necessary to confirm these findings and study their impact on important clinical outcomes, in particular on the incidence of apneas and on the improvement of comfort and sleep quality.

## Author Contributions

Florent Baudin contributed to data analysis, interpretation of results, and development of the manuscript. Hau-Tieng Wu conducted all NRR analysis, contributed in the interpretation of results, and participated in the development of the manuscript. Alice Bordessoule contributed to the recordings, data analysis, and revision of the manuscript. Jennifer Beck contributed to the data analysis, interpretation of results, and revisions of the manuscript. Philippe Jouvet contributed to the interpretation of results and revision of the manuscript. Martin G. Frasch contributed to the conception and design of the study, data analysis, interpretation of results, and development of the manuscript. Guillaume Emeriaud contributed to the conception and design of the study, data recordings, data analysis, interpretation of results, and development of the manuscript.

## Conflict of Interest Statement

Florent Baudin, Hau-Tieng Wu, Alice Bordessoule, Philippe Jouvet, Martin G. Frasch, and Guillaume Emeriaud have no commercial or financial relationships that could be construed as a potential conflict of interest. Jennifer Beck has been reimbursed by Maquet Critical Care (Solna, Sweden) for attending several conferences; Jennifer Beck has participated as a speaker in scientific meetings or courses organized and financed by Maquet Critical Care; Jennifer Beck, through Neurovent Research, serves as a consultant to Maquet Critical Care. The following disclosure was agreed upon by University of Toronto, Sunnybrook Health Sciences Centre, St-Michael’s Hospital and the REBs of Sunnybrook and St-Michael’s to resolve conflicts of interest: “Dr. Beck has made inventions related to neural control of mechanical ventilation that are patented. The patents are assigned to the academic institution(s) where inventions were made. The license for these patents belongs to Maquet Critical Care. Future commercial uses of this technology may provide financial benefit to Dr. Beck through royalties. Dr. Beck owns 50% of Neurovent Research Inc. (NVR). NVR is a research and development company that builds the equipment and catheters for research studies. NVR has a consulting agreement with Maquet Critical Care.” St-Michael’s Hospital has a research agreement with Maquet Critical Care AB (Solna, Sweden) and receives royalty and overhead from this agreement.
